# Some Virulence-Associated Genes of *Proteus* Isolates Could Predict Antibiotic Susceptibility and Even Infection Source

**DOI:** 10.1155/ijm/6022851

**Published:** 2025-11-10

**Authors:** Narges Jafari, Roya Ahmadrajabi, Omid Tadjrobehkar

**Affiliations:** ^1^Department of Medical Bacteriology & Virology, Afzalipour School of Medicine, Kerman University of Medical Sciences, Kerman, Iran; ^2^Gastroenterology and Hepatology Research Center, Institute of Basic and Clinical Physiology Sciences, Kerman University of Medical Sciences, Kerman, Iran

**Keywords:** antibiotic resistance, ESBLs, *Proteus*, virulence-associated genes

## Abstract

**Background:**

In the present study, the probable association of virulence-associated genes (VAGs) with antibiotic resistance and also sample sources in *Proteus* isolates was investigated.

**Methods:**

Then, 91 *Proteus mirabilis* and nine *Proteus vulgaris* were used in this study. The disk diffusion method was used in order to perform an antibiotic susceptibility assessment. A combination double-disc synergy test was used for the evaluation of extended-spectrum *β*-lactamases. Eight VAGs were investigated by polymerase chain reaction (PCR) method. ERIC-PCR fingerprinting was also performed for *P. mirabilis* isolates.

**Results:**

Maximum frequency of resistance was detected against trimethoprim–sulfamethoxazole combination in *P. mirabilis* isolates and against cefalexin in *P. vulgaris* isolates. Then, 6% of isolates were multidrug-resistant (MDR) and all were *P. mirabilis.* Community-acquired (CA) isolates were more virulent than hospital-acquired (HA) isolates. The *zapA* (98%) and *atfA* (77%) were the most common and less common VAGs, respectively. The study findings showed that *mrpA* and *atfA* genes were predictors of sensitivity to some antibiotic agents. The *rsbA* gene could also be similarly used in order to distinguish CA isolates from HA isolates.

**Conclusions:**

Higher virulence potential of CA isolates in comparison to the HA isolates is suggested. Amikacin, tobramycin, and meropenem were introduced as the most effective antibiotics against *Proteus* isolates. Our finding primarily introduced some VAGs as biomarkers for predicting antibiotic susceptibility and also in order to differentiate *Proteus* isolates. However, it has to be confirmed through complementary studies later.

## 1. Introduction

*Proteus mirabilis* and *Proteus vulgaris* were reported as the most frequent opportunistic pathogenic species of *Proteus* that were isolated from clinical samples [1]. *Proteus* species are involved in different types of infections including urinary tract infection (UTI), bacteremia, indwelling catheter-associated infections, wound infections, and also localized lesions in elderly and diabetic persons [2–4]. *Proteus* is also a major cause of nosocomial infections, especially catheter-associated urinary tract infections (CAUTIs) [5, 6].

A massive storage of virulence-associated genes (VAGs) was detected in the *Proteus* species. Diverse gene clusters express different adhesins (17 fimbriae operons) in the *Proteus* species [7]. Among them, mannose-resistant/*Proteus*-like (MR/P) fimbriae, *Proteus mirabilis* fimbriae (PMF), and *Proteus mirabilis* P-like pili (PMP) are the most studied, and they play critical roles in the colonization of *P. mirabilis* in the uroepithelium of humans [8].

MR/P fimbria is composed of two structural subunits, with the main subunit encoded by the *mrpA* gene and *mrpBEFG* encoding other subunits. The MR/P fimbriae are involved in the colonization of the urinary tract and also biofilm formation, especially in UTI cases [4, 9]. PMF is another fimbria of *P. mirabilis*, which is encoded by the *pmfACDEF* operon and is involved in the colonization of the bacterium in the kidney and bladder epithelium [10]. Uroepithelial cell adhesin (UCA) is another fimbria of the *Proteus* genus that is optimally expressed at the ambient temperature (23°C), and it facilitates the attachment of the *Proteus* species to the glycolipid molecules of the human uroepithelium under *in vitro* conditions [9, 10].

Several factors are associated with the destruction of the host tissues by *Proteus* species, including toxins, lipopolysaccharide (LPS), vigorous motility, swarming, and proteolytic enzymes [11, 12]. HpmA and HlyA are the two most important hemolysins of the *Proteus* genus. The *hpm* locus contained two associated genes, *hpmA*, which is a Ca2+-dependent pore-forming toxin, and *hpmB*, which is a toxin activator [13]. HpmA has a critical role in UTI pathogenesis [14] and also in biofilm formation [15].

The *fliL* gene is a member of the *fliLMNOPQR* locus that encodes a basal body protein of the flagellum [16]. The *fliL* has a critical role in the control of swimming and swarming behavior of *P*. *mirabilis,* and it also stimulates the expression of the *zapA* and *hpmA* genes [17]. The RsbA is a protein sensor of *P. mirabilis* and is encoded by the *rsbA* gene. The regulatory activities of the *rsbA* gene on swarming motility and biofilm formation have been reported recently [13, 14]. The *zapA* gene encodes a zinc metalloprotease of *P. mirabilis* with multifunctional properties. ZapA protease cleaves IgA and complement components of the human serum and protects bacteria against the host immune responses [5, 13].

Antibiotic resistance continuously has been regarded as a major global problem in health systems from the beginning of the antibiotic era (1950s) till now. It is suggested that antibiotic–resistance genes (ARGs) have evolved in the surrounding environment even before upcoming new antibiotics developed [18]. *Enterobacteriaceae* members such as *Proteus* are known as important antibiotic-resistant human pathogens. *Proteus* species are intrinsically resistant to polymyxin antibiotics, ampicillin, tetracycline, and nitrofurantoin, and also reduced susceptibility to other agents has been reported repeatedly [1, 19]. In the present study, we first tried to investigate the antibiotic susceptibility profile of *Proteus* species, and also, a detailed arsenal of VAGs has been detected. In the next step, the association between antibiotic resistance and VAGs has been analyzed. We also tried to find logical relationships between antibiotic resistance and VAGs.

## 2. Material and Methods

### 2.1. Specimen Collection

In the present study, some suspected *Proteus* isolates were cultivated by laboratory practitioners from hospitalized and outpatient individuals referred to different laboratories and medical centers affiliated with Kerman University of Medical Sciences. *Proteus* isolates were obtained from different samples (urine, surgical wounds, and burning wounds). They were transferred immediately to the bacteriology laboratory of Afzalipour School of Medicine for confirmatory identification and also species differentiation. Finally, 100 confirmed bacterial isolates (91 *Proteus mirabilis* and 9 *Proteus vulgaris*) were used in all experiments. The isolates that were isolated immediately after admission to the hospitals or within the first 48 h of hospitalization were referred to as community-acquired (CA), and the isolates that were obtained after 48 h of the hospitalization period were regarded as hospital-acquired (HA) [20, 21].

### 2.2. Culture and Identification

*Proteus* isolates were identified by standard biochemical and molecular tests [22, 23]. The isolated bacteria were stored in trypticase soy broth enriched with 20% glycerol at −70°C for subsequent use.

### 2.3. Antimicrobial Susceptibility Test

Disk diffusion method was used for antibiotic susceptibility assessments of *Proteus* isolates according to the guidelines of the Clinical and Laboratory Standards Institute (CLSI) [24].

Then, 20 antibiotic discs from the following classes: *β*-lactams including amoxicillin (25 *μ*g), cefalexin (30 *μ*g), cefuroxime (30 *μ*g), cefoxitin (30 *μ*g), ceftazidime (30 *μ*g), ceftriaxone (30 *μ*g), cefotaxime (30 *μ*g), cefepime (30 *μ*g), aztreonam (30 *μ*g), imipenem (10 *μ*g), and meropenem (10 *μ*g); fluoroquinolones including norfloxacin (10 *μ*g) and ciprofloxacin (5 *μ*g); aminoglycosides including tobramycin (10 *μ*g), gentamicin (10 *μ*g), amikacin (30 *μ*g), and trimethoprim/sulfamethoxazole (1.23/25.75 *μ*g) as folate synthesis inhibitors were used in order to assess antibiotic susceptibility. Antibiotic discs were provided by Padtan-Teb.Co.(Iran). *E. coli* ATCC 25922 was used for quality control of the disk diffusion method. All experiments were performed in triplicate.

### 2.4. Extended Spectrum *β*-Lactamase (ESBL) Screening and Confirmatory Test

Isolates showed primary resistance against aztreonam, ceftazidime, and cefotaxime in the screening disk diffusion method and were regarded as ESBL producers. ESBL-producing isolates were confirmed using two pairs of antibiotic disks: ceftazidime (30 *μ*g), ceftazidime–clavulanic acid (30 *μ*g/10 *μ*g), cefotaxime (30 *μ*g), and cefotaxime–clavulanic acid (30 *μ*g/10 *μ*g) through the combination double-disc synergy test (CDDST) according to CLSI guidelines [24, 25].

### 2.5. Bacterial Cell Lysate Preparation and DNA Extraction

In summary, a loopful of an overnight culture of bacterial isolates was inoculated into the microtubes including 500 *μ*L of distilled water. The microtubes were heated in a water bath at 100°C for 10 min. In the next step, the sample was centrifuged (12,000 rpm for 5 min). After centrifugation, the supernatant was separated and kept at −70°C.

### 2.6. Polymerase Chain Reaction and Primer List

The eight VAGs (*rsbA*, *mrpA*, *zapA*, *atfA*, *hpmA*, *pmfA*, *ucaA*, and *fliL*) were investigated by PCR method using a Biometra thermocycler (Germany) and specific primers (purchased from Bioneer, South Korea). The primer sequences are shown in Table 1.

PCR products were separated by gel electrophoresis using a 1.5% agarose gel alongside 100-bp DNA ladder.

### 2.7. Enterobacterial Repetitive Intergenic Consensus (ERIC) PCR Fingerprinting

The following pair of recommended primers 5⁣′ ATGTAAGCTCCTGGGGATTCAC 3⁣′ and 5⁣′ AAGTAAGTGACTGGGGTGAGCG 3⁣′ was used in fingerprinting of *P. mirabilis* isolates by ERIC-PCR technique [29]. After separation of PCR products by gel electrophoresis, band sizes were recorded in the Excel software datasheets, and finally, band size patterns were analyzed by NTSYSpc v2-10e software, and a dendrogram was created through unweighted pair group method with arithmetic mean (UPGMA) by using Dice coefficient, and 95% genetic distance was used for allocation of isolates in different clusters.

### 2.8. Statistical Analysis

Data analysis was performed using SPSS 26 statistical software. Chi-square and Fisher's exact test were used to analyze the association between variables. Binary logistic regression analysis was used to evaluate the predictive role of VAGs for resistance or susceptibility to different antibiotic agents. All experiments were performed in triplicate and means were used. The *p* value ≤ 0.05 was considered significant.

## 3. Results

### 3.1. Distribution of Bacterial Isolates in Different Samples

Totally, 60 out of 100 isolates were regarded as HA; 54 (90%) of them were *P. mirabilis* and 6 (10%) isolates were confirmed as *P. vulgaris*. The prevalence of *P. mirabilis* and *P. vulgaris* in CA isolates was 37 (92.5%) and 3 (7.5%), respectively. The majority of *P. mirabilis* isolates, 82 (90.1%), were cultivated from urine samples; three (3.3%) isolates were cultivated from surgical wounds and six (6.6%) isolates were obtained from burning wounds. Eight (88.9%) out of nine *P. vulgaris* isolates were cultivated from urine samples and only one isolate (11.1%) was obtained from a surgical wound.

### 3.2. Antibiotic Susceptibility

Data collected from antibiotic susceptibility assessment showed various frequencies of resistance against the following antibiotic agents: *β*-lactam antibiotics including amoxicillin (31%), cefalexin (42%), cefuroxime (36%), cefoxitin (16%), ceftriaxone (19%), cefotaxime (26%), ceftazidime (10%), cefepime (4%), meropenem (10%), imipenem (12%), and aztreonam (7%), trimethoprim–sulfamethoxazole (67%) as a folate synthesis inhibitor, aminoglycosides including amikacin (6%), tobramycin (10%), and gentamicin (21%), and fluoroquinolones including norfloxacin (20%) and ciprofloxacin (25%).

**Figure 1:**
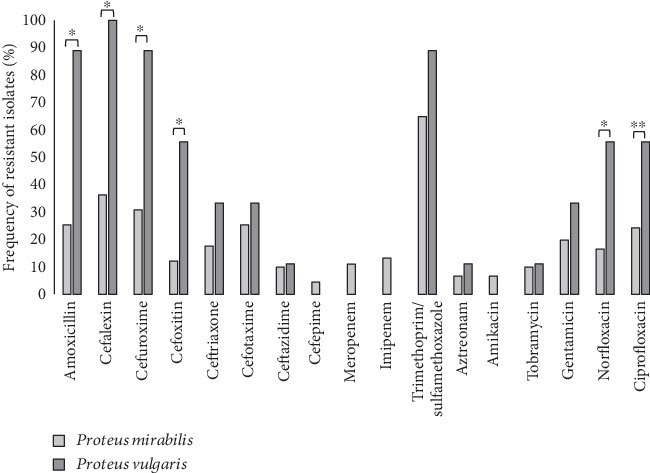
Frequency (%) of resistance against different antibiotic agents in two *Proteus* species. ⁣^∗^*p* ≤ 0.005 and ⁣^∗∗^*p* ≤ 0.05.

Maximum antibiotic resistance was detected against cefalexin (100%) in *P. vulgaris* isolates, and maximum resistance was detected against trimethoprim–sulfamethoxazole (64.8%) in *P. mirabilis*. Minimum antibiotic resistance was detected against cefepime (4.4%) and amikacin (6.6%) in *P. mirabilis.* Resistance against cefepime, amikacin, imipenem, and meropenem was not detected in *P. vulgaris* isolates (Figure 1).

Isolates that were not susceptible to three antibiotic agents from different classes were regarded as multidrug-resistant [19]. Totally, six isolates (6%) were MDR, of which 5 were *P. mirabilis* and only 1 *P. vulgaris* isolate was MDR. No extremely drug-resistant (XDR) isolate was detected in our study. There were not significant differences between the frequency of MDR isolates among HA isolates and CA isolates.

Variable frequency of resistance was detected against different antibiotic agents among two studied species (Figure 1). Higher frequency of resistance against amoxicillin, cefalexin, cefuroxime, cefoxitin, norfloxacin, and ciprofloxacin was detected in *P. vulgaris* isolates in comparison to *P. mirabilis* isolates (Figure 1).

Data obtained from the double disk synergy test revealed that 12 (13.2%) of *P. mirabilis* isolates and one (11.1%) of *P. vulgaris* isolates were ESBL producers (totally 13% of *Proteus* isolates).

**Figure 2:**
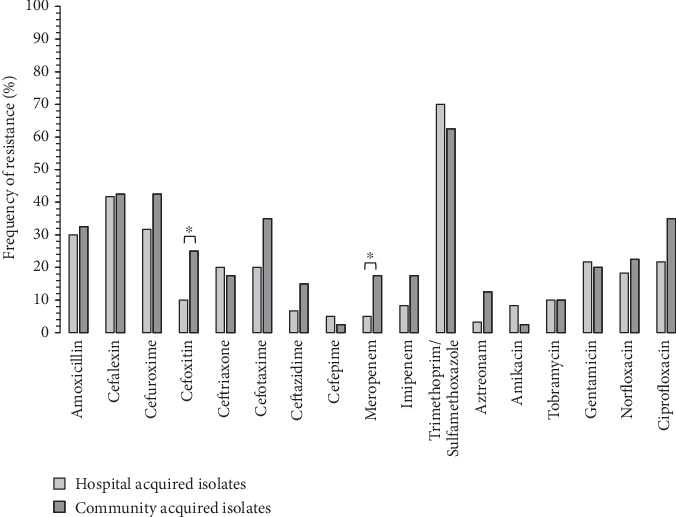
Frequency of antibiotic resistance in HA-isolates in comparison to the CA isolates. ∗*p* ≤ 0.05.

Pearson Chi-square analysis showed that resistance against cefoxitin (*p* = 0.021) and meropenem (*p* = 0.041) was significantly higher in CA isolates in comparison to the HA isolates. There were not observed significant differences regarding remaining antibiotic agents (Figure 2).

Fisher's exact test analysis showed that ESBL activity was significantly (*p* ≤ 0.05) more frequent in CA isolates (22.5% and 17.5%, respectively) in comparison to the HA isolates (6.7% and 5%, respectively).

### 3.3. Frequency of VAGs

Prevalence of studied VAGs among *Proteus* isolates was as follows: *rsbA* (93%), *mrpA* (82%), *zapA* (98%), *atfA* (77%), *hpmA* (97%), *pmfA* (86%), *ucaA* (88%), and *fliL* (97%).

**Figure 3:**
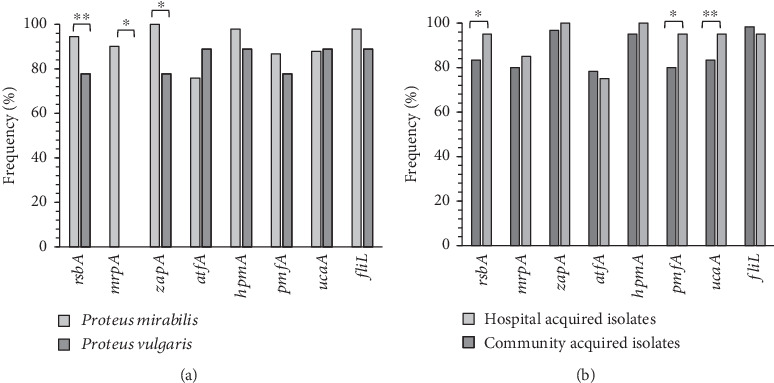
(a) Frequency (%) of the VAGs in two studied species. (b) Frequency (%) of the VAGs in HA-isolates than CA isolates. ⁣^∗^*p* ≤ 0.05 and ⁣^∗∗^*p* ≤ 0.07.

Statistical analysis showed that the distribution of the *zapA* and *mrpA* genes was significantly different (Fisher's exact test analysis, *p* ≤ 0.05) between two investigated *Proteus* species. The *mrpA* gene was detected only in *P. mirabilis* isolates (Figure 3).

Chi-square analysis revealed that the *rsbA* gene was more prevalent in CA isolates in comparison to the HA isolates (100% against 88.3%, *p* = 0.024). Similar results were detected for the *pmfA* gene (95% against 80%, *p* = 0.034). Data have been presented in Figure 3.

Collection of all of the studied VAGs was detected in 47 (47%) out of 100 isolates. Pearson Chi-square analysis confirmed that these highly virulent isolates were harvested significantly (*p* = 0.027) more from the community (60%) than from the hospital (37.3%). Antibiotic resistance against different antibiotic agents was not significantly (*p* ≤ 0.05) different in these isolates than in the others.

### 3.4. Association of VAGs With Antibiotic Resistance

Pearson Chi-square analysis showed that the *atfA* gene is significantly associated with sensitivity to tobramycin (*p* = 0.032) and amikacin (*p* = 0.044). Data have been presented in Table 2. The binary regression analysis revealed that the *atfA* gene is a predictor of sensitivity to amikacin (*p* = 0.043, odds ratio: 1.188) and tobramycin (*p* = 0.019, odds ratio: 2.209).

Data analysis (Pearson Chi-square) also revealed that the *mrpA* gene was significantly associated with susceptibility against amoxicillin (*p* = 0.013), cefoxitin (*p* = 0.027), and norfloxacin (*p* = 0.027). Data have been presented in Table 2. The binary regression analysis also revealed that the *mrpA* gene is a predictor of sensitivity to cefoxitin (*p* = 0.005, odds ratio: 3.262), norfloxacin (*p* = 0.015, odds ratio: 1.817), and amoxicillin (*p* = 0.021, odds ratio: 2.317).

### 3.5. Genotyping Trough ERIC-PCR Fingerprinting Method

**Figure 4:**
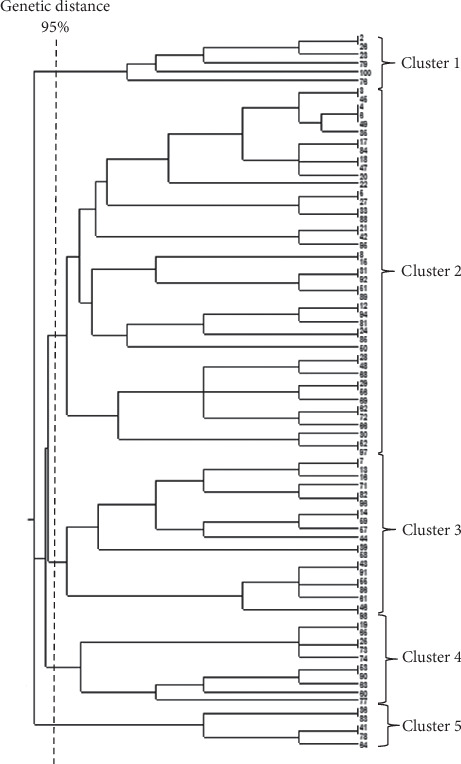
DNA fingerprinting by ERIC-PCR. Dendrogram allocated 82 *P. mirabilis* isolates in five clusters using UPGAMA method with Dice coefficient. The 95% genetic distance was used for the allocation of isolates in different clusters.

Only *P. mirabilis* isolates were typable by the ERIC-PCR method. Only 83 out of 91 *P. mirabilis* isolates were categorized in five clusters, and eight (8.8%) isolates were totally different from other isolates. Six isolates (6.6%) were placed in Cluster 1; Clusters 2 to 5 included 46 (50.5%), 16 (17.5%), 10 (11%), and 5 (5.5%) isolates, respectively (Figure 4). Statistical analysis did not show any significant association between different ERIC clusters and the sample or source type of isolates.

### 3.6. Frequency of Phenotypic Antimicrobial Resistance Among Different Clusters

Cluster 5 contains five CA urine isolates. These isolates were significantly different from other clusters regarding resistance against amoxicillin, cefalexin, cefoxitin, cefotaxime, ceftazidime, cefuroxime, and meropenem. Tobramycin and cefepime resistance were more frequent in Cluster 1 and Cluster 3, respectively (Table 3). Statistical analysis did not find any significant differences between other clusters regarding antibiotic resistance or virulence gene profile.

### 3.7. Frequency of VAGs Among Different Clusters

Frequency of *rsbA* gene was significantly different (Chi-square analysis, *p* = 0.001) among five ERIC clusters, and minimum frequency (60%) was detected among Cluster 5 isolates. Meanwhile, cumulative frequency of *rsbA* gene among other clusters was 96.2%. The *rsbA* gene was detected in all isolates in Clusters 1, 3, and 4, and 93.5% of isolates in Cluster 2 also had it.

## 4. Discussion

Uncontrolled use of antibiotic agents in various industries, including agriculture, animal husbandry, food-related industries, and also medicine during the last decades, has resulted in widespread evolution of antibiotic resistance in different bacterial pathogens [30]. In this context, reduced susceptibility of *Proteus* species to different antibiotic agents was also reported globally, and the spread of MDR and XDR strains has limited treatment options [1, 31].

In the present study, maximum resistance was detected against the trimethoprim-sulfamethoxazole combination in *P. mirabilis* isolates (64.8%). Mirzaei et al. also reported similar findings from a different geographic region of Iran recently [32]. The maximum resistance of *P. mirabilis* isolates against the trimethoprim–sulfamethoxazole combination was also reported from other countries [31, 33, 34].

Low frequency of resistance against different *β*-lactam antibiotics, aminoglycosides, and fluoroquinolones was observed in *P. mirabilis* isolates in the present study. Similar findings were also reported in a meta-analysis from Iran recently that support our findings [35]. However, increasing resistance against these antibiotic agents is also reported from other countries [31]. Low frequency of resistance against *β*-lactam antibiotics among *P. mirabilis* isolates in our study could also be explained by the low prevalence of ESBL-producing isolates (13%) among the investigated bacterial population. Similar findings were also reported by other researchers [36, 37]. Different frequencies of resistance against *β*-lactam antibiotics, fluoroquinolones, and aminoglycosides have been reported from different countries. It could be the result of different patterns of antibiotic prescription in various geographical regions worldwide, and horizontal transfer of ARGs within integrons, transposons, and plasmids between different Enterobacteriaceae members could be regarded as the major reasonable cause [31].

Our analysis showed a low resistance rate against amikacin, tobramycin, meropenem, and aztreonam in *Proteus* isolates. In this extent, high susceptibility to aminoglycoside antibiotics, except gentamicin, carbapenems, and aztreonam, was reported in other studies recently [29, 38–41]. Therefore, these antibiotics could be indicated as the most effective antibiotic agents against pathogenic *Proteus* isolates.

All the investigated VAGs except *atfA* were detected in more than 80% of the *Proteus* isolates. The *atfA* gene was detected only in 77% of isolates, and *zapA* (98%) had maximum frequency. Elhoshi et al. have also reported *zapA* (100%) and *atfA* (84%) as the VAGs with maximum and minimum frequency, respectively [42].

Data analysis showed that all of the studied VAGs were collectively detected in 47% of isolates. Thus, they could be assigned as highly virulent isolates, and the majority of them (60%) were CA. Therefore, the higher virulence potential of CA isolates in comparison to the HA isolates could be estimated. Similar findings were reported recently regarding other bacteria such as *Escherichia coli*, *Staphylococcus aureus*, and *Pseudomonas aeruginosa* [43–45]. It could logically explain that community isolated bacteria need a richer store of virulence factors for surviving among a very versatile community of bacterial and nonbacterial competitors in the surrounding environment in comparison to the HA isolates that live in an antimicrobial-rich environment of hospitals with a comparable limited number of competitors. Instead, HA isolates acquired more complex antibiotic-resistance mechanisms. Similar estimation has also been reported before by Radera et al. [43].

Association of some of the VAGs with susceptibility against different antibiotic agents was reported regarding *Proteus* isolates recently [14, 28]. In the present study, logistic regression analysis of data showed that the *atfA* gene was a predictor of sensitivity to amikacin and tobramycin. The *mrpA* gene was detected only in *P. mirabilis* isolates, and the predictive role of the *mrpA* gene for susceptibility to cefoxitin, norfloxacin, and amoxicillin was also detected by logistic regression analysis. Finally, it could be concluded that *atfA* and *mrpA* could be assigned as predictor VAGs for estimating antibiotic susceptibility.

Using qualitative morphologic methods such as the disk diffusion test in order to assess antibiotic susceptibility is popularized because of the simplicity and economic efficiency. But the results are not always trustable. On the other hand, molecular evaluation of antibiotic resistance through screening of the responsible genes, considering the versatile store of ARGs, is usually more expensive and time-consuming in comparison to the phenotypic methods. Therefore, we thought in some cases special VAGs could be introduced as suitable markers for the prognosis of antibiotic susceptibility. According to this, the *atfA* gene could be introduced as a genetic marker for simple and quick diagnosis of susceptibility against aminoglycoside antibiotics such as amikacin and tobramycin. The *mrpA* gene could be used similarly for the estimation of susceptibility to cefoxitin, norfloxacin, and amoxicillin in *Proteus* isolates. However, it has to be evaluated with more detail through future studies.

Based on the study finding, the *rsbA* gene was detected in all of the CA-isolates. Therefore, it may be introduced for differentiation of HA *Proteus* isolates from CA isolates. The *rsbA* gene involved regulation of chemotactic motility and swarming motility, and it also controls expression of other VAGs [46, 47]. Thus, the critical role of the *rsbA* gene in the natural survival of *Proteus* species, especially in the surrounding natural environment, and also the commensal behavior of *Proteus* species in the intestinal tract of animals and humans could be concluded. Therefore, the higher frequency of this gene in CA isolates in comparison to the HA isolates could be logically acceptable.

ERIC-PCR fingerprinting revealed high diversity among studied *P. mirabilis* isolates. In such a way that, 53 different patterns were detected for 83 isolates. Finally, five patterns were repeated among the studied isolates at 95% genetic distance. Hence, studied isolates were allocated in 5 different clusters (95% confidence interval for genetic distance). ERIC-PCR is known as a relatively simple and not-expensive method that has some limitations such as low reproducibility; however, it has acceptable outcomes, especially in epidemiological studies. In the present study, *Proteus* isolates were collected from different sources and also diverse samples. Therefore, such high diversity was expected and is acceptable.

## 5. Conclusion

The present study is one of the few reports that proposed using VAGs for differentiation of *Proteus* isolates from each other and also antibiotic susceptibility estimation. We suggest that *atfA and mrpA* genes are marker genes for sensitivity of *Proteus* species to antibiotic agents. Moreover, the *rsbA* gene could be introduced as a genetic marker in order to distinguish CA isolates from HA *Proteus* isolates. Meanwhile, these have to be investigated in a bigger, multicenter bacterial population later. In the present study, only gene presence/absence was screened through PCR without functional assays. Hence, some confirmatory tests such as VAG and ARG expressions in association with antibiotic resistance are proposed through future studies that would confirm our findings. We believe that the conduction of the same studies on more VAGs in association with ARGs could be beneficial in finding a probable direct association between VAGs and antibiotic resistance. It may provide new insights for designing future diagnostic protocols and also treatment strategies. We had some limitations that have to be explained. We do not have access to patient clinical characteristics, which might be helpful in data analysis.

## Figures and Tables

**Table 1:** The primer sequences that were used for screening of VAGs through PCR amplification process.

**Targeted genes**		**Primer (5**⁣′**→3**⁣′**)**	**Product size (bps)**	**References**
*rsbA*	F	TTGAAGGACGCGATCAGACC	467	[26]
	R	ACTCTGCTGTCCTGTGGGTA		
*zapA*	F	TATCGTCTCCTTCGCCTCCA	332	[27]
	R	TGGCGCAAATACGACTACCA		
*mrpA*	F	GAGCCATTCAATTAGGAATAATCCA	648	[27]
	R	AGCTCTGTACTTCCTTGTACAGA		
*hpmA*	F	CCAGTGAATTAACGGCAGGT	654	[28]
	R	CGTGCCCAGTAATGGCTAAT		
*fliL*	F	CTCTGCTCGTGGTGGTGTCG	770	[17]
	R	GCGTCGTCACCTGATGTGTC		
*ucaA*	F	GTAAAGTTGTTGCGCAAAC	560	[28]
	R	TTGAGCCACTGTGGATACA		
*pmfA*	F	CAAATTAATCTAGAACCACTC	618	[28]
	R	ATTATAGAGGATCCCTTGAAGGTA		
*atfA*	F	CATAATTTCTAGACCTGCCCTAGCA	382	[28]
	R	CTGCTTGGATCCGTAATTTTTAACG		

Abbreviations: F, forward primer; R, reverse primer.

**Table 2:** Frequency of virulence-associated genes in antibiotic-resistant isolates in comparison to the susceptible isolates.

**Antibiotics**	**R or S (** **n** **)**	**Virulence-associated genes, ** **n** **(%)**							
		***rsbA***	***mrpA***	***zapA***	***atfA***	***hpmA***	***pmfA***	***ucaA***	***fliL***
Amoxicillin	**R** (31)	27 (87.1)	**21 (67.7)**	29 (93.5)	24 (77.4)	30 (96.8)	25 (80.6)	27 (87.1)	30 (96.8)
	**S** (69)	66 (95.7)	**61 (88.4)**	69 (100)	53 (76.8)	67 (97.1)	61 (88.4)	61 (88.4)	67 (97.1)

Cephalexin	**R** (42)	38 (90.5)	32 (76.2)	40 (95.2)	30 (71.4)	40 (95.2)	35 (83.3)	36 (85.7)	40 (95.2)
	**S** (58)	55 (94.8)	50 (86.2)	58 (100)	47 (81.0)	57 (98.3)	51 (87.9)	52 (89.7)	57 (98.3)

Cefuroxime	**R** (36)	33 (91.7)	27 (75)	34 (94.4)	28 (77.8)	34 (94.4)	30 (83.3)	33 (91.7)	34 (94.4)
	**S** (64)	60 (93.8)	55 (85.9)	64 (100)	49 (77.6)	63 (98.4)	56 (87.5)	55 (85.9)	63 (98.4)

Cefoxitin	**R** (16)	15 (93.8)	**10 (62.5)**	15 (93.8)	12 (75.0)	15 (93.8)	15 (93.8)	13 (81.3)	16 (100)
	**S** (84)	78 (92.9)	**72 (85.7)**	83 (98.8)	65 (77.4)	82 (97.6)	71 (84.5)	75 (89.3)	81 (96.4)

Ceftriaxone	**R** (19)	17 (89.5)	15 (78.9)	18 (94.7)	14 (73.7)	18 (94.7)	16 (84.2)	18 (94.7)	19( 100)
	**S** (81)	76 (93.8)	68 (82.87)	80 (98.8)	63 (77.8)	79 (97.5)	70 (86.4)	70 (86.4)	78 (96.3)

Cefotaxime	**R** (26)	24 (92.3)	22 (84.6)	25 (96.2)	19 (73.1)	24 (92.3)	23 (88.5)	24 (92.3)	25 (96.2)
	**S** (74)	69 (93.2)	60 (81.1)	73 (98.6)	58 (78.4)	73 (98.6)	63 (85.1)	64 (86.5)	72 (97.3)

Ceftazidime	**R** (9)	9 (100)	9 (100)	9 (100)	5 (55.6)	9 (100)	9 (100)	7 (77.8)	9 (100)
	**S** (91)	84 (92.3)	73 (80.2)	89 (97.8)	72 (79.1)	88 (96.7)	77 (84.6)	81 (89)	88 (96.7)

Cefepime	**R** (4)	4 (100)	3 (75.0)	4 (100)	4 (100)	4 (100)	4 (100)	4 (100)	4 (100)
	**S** (96)	82 (92.7)	79 (82.3)	94 (97.9)	73 (76.0)	93 (96.9)	82 (85.4)	84 (87.5)	93 (96.9)

Meropenem	**R** (10)	10 (100)	10 (100)	10 (100)	7 (70.0)	10 (100)	10 (100)	9 (90.0)	10 (100)
	**S** (90)	83 (92.2)	72 (80)	88 (97.8)	70 (77.8)	87 (96.7)	76 (84.4)	79 (78.8)	78 (96.7)

Imipenem	**R** (12)	12 (100)	12 (100)	12 (100)	7 (66.7)	12 (100)	12 (100)	10 (83.3)	12 (100)
	**S** (88)	81 (92)	70 (79.5)	86 (97.7)	69 (78.4)	86 (96.6)	74 (84.1)	78 (88.6)	85 (96.6)

Co-trimoxazole	**R** (67)	63 (94)	53 (79.1)	65 (97)	52 (77.6)	64 (95.5)	58 (86.6)	60 (89.6)	64 (95.5)
	**S** (33)	30 (90.9)	29 (87.9)	33 (100)	25 (75.8)	33 (100)	28 (84.8)	28 (84.8)	33 (100)

Aztreonam	**R** (7)	7 (100)	5 (71.4)	7 (100)	7 (100)	6 (85.7)	7 (100)	6 (85.7)	7 (100)
	**S** (93)	86 (92.5)	77 (82.8)	91 (97.8)	70 (75.3)	91 (97.8)	79 (84.9)	82 (88.2)	90 (96.8)

Amikacin	**R** (6)	6 (100)	6 (100)	6 (100)	**3 (50.0)**	6 (100)	6 (100)	5 (83.3)	6 (100)
	**S** (94)	87 (92.6)	76 (80.9)	92 (97.9)	**74 (78.7)**	91 (96.8)	80 (85.1)	83 (88.3)	91 (96.8)

Tobramycin	**R** (10)	9 (90.0)	7 (70.0)	9 (90.0)	**5 (50.0)**	10 (100)	9 (90.0)	8 (80.0)	9 (90.0)
	**S** (90)	84 (93.3)	85 (83.3)	89 (98.9)	**72 (80.0)**	87 (96.7)	77 (85.6)	80 (88.9)	88 (97.8)

Gentamicin	**R** (21)	19 (90.5)	17 (81)	19 (90.5)	15 (71.4)	20 (95.2)	18 (85.7)	19 (90.5)	20 (95.5)
	**S** (79)	74 (93.7)	65 (82.3)	79 (100)	62 (78.5)	77 (97.5)	68 (86.1)	69 (87.3)	77 (97.5)

Norfloxacin	**R** (20)	19 (95)	**13 (65.0)**	19 (95.0)	14 (70.0)	20 (100)	19 (95.0)	13 (65)	18 (90.0)
	**S** (80)	74 (92.5)	**69 (86.3)**	7 9(98.8)	63 (78.8)	77 (96.3)	67 (83.8)	72 (90.0)	79 (98.8)

Ciprofloxacin	**R** (27)	25 (92.6)	20 (74.1)	26 (96.3)	18 (66.7)	27 (100)	26 (96.3)	23 (85.2)	25 (92.6)
	**S** (73)	68 (93.2)	62 (84.9)	72 (98.6)	59 (80.8)	70 (95.9)	60 (82.2)	65 (89.0)	72 (98.6)

*Note:* Bold *n* (%): Pearson Chi-square analysis showed significant association (*p* ≤ 0.05). Trimethoprim–sulfamethoxazole combination referred as co-trimoxazole.

Abbreviations: R, resistant; S, susceptible.

**Table 3:** Frequency of antibiotic resistance among different ERIC clusters.

**Antibiotic agents**	**C1** **6 (100%)**	**C2** **46 (100%)**	**C3** **16 (100%)**	**C4** **10 (100%)**	**C5** **5 (100%)**
Amoxicillin	3 (50)	12 (26.1)	1 (6.3)	2 (20)	3 (60)
Cefalexin	4 (66.7)	14 (30.4)	6 (37.5)	3 (30)	**4 (80)**
Cefotaxime	2 (33.3)	9 (19.6)	3 (18.8)	3 (30)	**4 (80)**
Ceftazidime	2 (33.3)	1 (2.2)	0 (0)	2 (20)	**2 (40)**
Cefuroxime	3 (50)	11 (23.9)	5 (31.3)	3 (30)	**4 (80)**
Cefoxitin	1 (16.7)	4 (7.8)	0 (0)	2 (20)	**2 (40)**
Cefepime	0 (0)	1 (2.2)	**2 (12.5)**	0 (0)	0 (0)
Meropenem	1 (16.7)	3 (6.5)	1 (6.3)	2 (20)	**2 (40)**
Tobramycin	**2 (33.3)**	5 (10.9)	0 (0)	0 (0)	0 (0)

*Note:* Bold *n* (%) means, Pearson Chi-square analysis showed significant association (*p* ≤ 0.05).

## Data Availability

The datasets used and/or analyzed during the current study are available from the corresponding author on reasonable request.
